# Current concepts in anterior glenohumeral instability: diagnosis and treatment

**DOI:** 10.1051/sicotj/2021048

**Published:** 2021-09-14

**Authors:** Daniel Moya, Nuri Aydin, Nobuyuki Yamamoto, Juan Pablo Simone, Paul Patiño Robles, Graham Tytherleigh-Strong, Bruno Gobbato, Erica Kholinne, In-Ho Jeon

**Affiliations:** 1 Department of Orthopedic Surgery, Hospital Británico de Buenos Aires C1280 AEB Buenos Aires Argentina; 2 Istanbul University-Cerrahpasa, Cerrahpasa Faculty of Medicine, Department of Orthopedics and Traumatology 34098 Istanbul Turkey; 3 Department of Orthopaedic Surgery, Tohoku University School of Medicine 980-8575 Sendai Japan; 4 Department of Orthopaedic Surgery, Hospital Alemán de Buenos Aires C1118 AAT Buenos Aires Argentina; 5 Artrocentro Santa Cruz de la Sierra Bolivia; 6 Division of Orthopaedics, Addenbroke’s Hospital, Cambridge University Hospitals Trust CB2 0QQ Cambridge United Kingdom; 7 Department of Orthopaedic Surgery, Hospital Sao Jose Jaraguá do Sul SC 89251-830 Brazil; 8 Faculty of Medicine, Universitas Trisakti, Department of Orthopaedic Surgery, St Carolus Hospital 10440 Jakarta Indonesia; 9 Department of Orthopaedics, University of Ulsan, College of Medicine, Asan Medical Center 05505 Seoul Korea

**Keywords:** Shoulder, Instability, Glenohumeral, Latarjet, Bankart

## Abstract

The glenohumeral joint is the most dislocated articulation, accounting for more than 50% of all joint dislocations. The reason behind shoulder instability should be investigated in detail for successful management, and the treatment plan should be individualized for all patients. Several classification systems have been proposed for glenohumeral instability. A physical exam is mandatory no matter what classification system is used. When treating patients with anterior shoulder instability, surgeons need to be aware of the critical size of the bone loss, which is commonly seen. The glenoid track concept was clinically adopted, and the measurement of the glenoid track for surgical decision-making is recommended. Detailed assessment of existing soft tissue injury to the labrum, capsule, glenohumeral ligaments, and rotator cuff is also mandatory as their presence influences the surgical outcome. Rehabilitation, arthroscopic repair techniques, open Bankart procedure, capsular plication, remplissage, Latarjet technique, iliac crest, and other bone grafts offer the surgeon different treatment options according to the type of patient and the lesions to be treated. Three-dimensional (3D) technologies can help to evaluate glenoid and humeral defects. Patient-specific guides are low-cost surgical instruments and can be used in shoulder instability surgery. 3D printing will undoubtedly become an essential tool to achieve the best results in glenohumeral instability surgery.

## Introduction

The shoulder is the most dislocated joint, which accounts for more than 50% of all joint dislocations [1]. Its capability of reaching a wide range of motion makes it susceptible to instability. The shoulder’s stability is provided by some static and dynamic factors, including the labrum, ligaments, and surrounding muscles. Instability can develop due to a pathology that comprises one or more of these stabilizing factors. Trauma is the most common reason leading to shoulder instability [2]. The traumatic shoulder dislocations are generally classified according to the direction of the instability as anterior, posterior, inferior. Anterior dislocation constitutes most of the shoulder dislocations with a rate of more than 95%. The rate of posterior dislocation is reported around 2–4%, while inferior dislocation is reported as low as 0.5% of all shoulder dislocations [3]. Although shoulder trauma is the most common triggering factor, shoulder instability can exist without significant trauma due to soft tissue abnormalities or impaired muscular function. Atraumatic instabilities are generally multidirectional, and their management differs from traumatic instabilities.

A comprehensive history-taking and examination are crucial to understanding the mechanism behind the failure. Overlooking of a contributing factor can result in unsuccessful management of the instability. Besides addressing the instability, the accompanying pathologies should also be considered for definitive management of the problem. Besides a thorough examination, the imaging modalities also have an important place to reveal the pathology behind the instability and quantify the problem to offer the appropriate solution. Radiology and computed tomography provide important insight into the bony structure and the amount of bone loss. Magnetic resonance imaging gives a chance for a detailed evaluation of the soft tissue structures to determine the possible problems, which should also be addressed during the treatment plan.

A shoulder instability treatment options include a wide range of interventions from rehabilitative measures to surgical repair or reconstruction of the disrupted mechanisms. Nonoperative treatment is a good option in multidirectional instability and the first episode of instability without associated risk factors [4]. When there are high-risk factors for recurrence in the first episode (such as young age, male, athletic activity, and presence of bone deficiency) or in case of recurrent instability, the pathology might be amenable to repair if no significant bone loss exists. However, in case of a significant disruption in bony architecture and young collision or contact athletes, more detailed and complex procedures might be required to reconstruct the shoulder girdle [5, 6]. Since the chance of bony disruption can increase with subsequent dislocations [7], the timing of the intervention is also important for successful management as the success of the treatment diminishes when more complex interventions are required [4]. Shoulder rehabilitation is also important to regain the sensorimotor function of the shoulder and the strength of the dynamic stabilizers [8]. Recurrence of the instability after conservative or surgical treatment might be inevitable without proper rehabilitation [8].

The reason behind shoulder instability should be investigated in detail for successful management, and the treatment plan should be individualized for all patients. The possible risk factors should also be determined to develop successful prevention and treatment algorithms [4–7, 9, 10].

## Biomechanics of shoulder instability

When treating patients with anterior shoulder instability, surgeons need to be aware of the critical size of the bone loss (glenoid defect and Hill-Sachs lesion), which are commonly seen. It is widely accepted that Bankart repair is a gold standard procedure, and there have been many reports describing the excellent clinical outcomes [11, 12]. However, a large bone loss has been demonstrated to cause postoperative shoulder instability. Several biomechanical studies have demonstrated which size of bone loss affects stability. Bone grafting is required if the glenoid bone loss is more than 25% of the glenoid width [13–15]. When we consider the critical size of the Hill-Sachs lesion, we must consider the glenoid bone loss simultaneously because engagement always occurs between a Hill-Sachs lesion and the glenoid rim. A new concept of the glenoid track was proposed in 2007 [16]. Using this concept, we can evaluate whether a large Hill-Sachs lesion is an “on-track” or “off-track” lesion and consider both bone loss at the same time. This concept has been widely used. The mean glenoid track width is demonstrated to be 83% of the glenoid width [17]. However, this width seems to be affected by the range of shoulder motion because the glenoid track is defined as the contact of the glenoid on the humeral head. An MRI study including healthy volunteers was performed to clarify the relationship between the range of motion and the glenoid track [18]. Results demonstrated that the greater the horizontal extension angle in abduction and external rotation, the smaller the glenoid track width. Individualized glenoid track width can be obtained by measuring the active horizontal extension angle in the sitting position.

Di Giacomo et al. [5] stated that engaging and non-engaging lesions are consistent with the concept of glenoid track described by Yamamoto et al. [16]. They are complementary concepts in that they evaluate the interaction of bipolar bone loss during shoulder function [5].

The glenoid track concept was clinically adopted, and the measurement of the glenoid track for surgical decision-making is recommended [6]. Ninety-four patients were assessed to clarify the clinical outcomes of patients surgically treated based on this concept [6]. The overall recurrence rate in patients treated based on the glenoid track concept was 4.3% at a 2-year follow-up. These results indicate the validity of the glenoid track concept in surgical decision-making to prevent recurrent instability after surgery.

In a case with an “on-track” lesion and less than 25% glenoid defect, arthroscopic Bankart repair alone is enough. In a case with an “off-track” lesion and less than 25% glenoid defect, we should treat such a Hill-Sachs lesion by remplissage procedure. In a case with an “on-track” lesion and more than 25% glenoid defect, we should treat a large glenoid bone loss by bone grafting such as the Latarjet procedure [5, 6, 19]. Even in the case of an “off-track” lesion and a more than 25% glenoid defect, bone grafting can be chosen because bone grafting converts an “off-track” lesion to an “on-track” lesion. We do not need to treat the Hill-Sachs lesion in this case. The bone graft will widen the glenoid track to such an extent that the Hill-Sachs lesion cannot go off track [5].

In addition to bone injuries, other factors such as the patient’s age, presence of laxity, type of sport, degree of sport participation must be considered.

## Classification and clinical examination

Several classification systems have been proposed for glenohumeral instability. [20–23] The patient’s signs and symptoms are gathered so that a pattern can be established to classify their condition. Once classified, the treatment strategy should follow accordingly in a systematic way or with an algorithmic approach. Because there are so many variables to consider before treating a patient with instability (etiology, instability direction, laxity, patient age, activity, expectations, bone loss), there is no perfect classification that will fit every case [24]. A critical assessment of the patient’s history, physical exam, and imaging studies must be done so the surgeon can provide optimal treatment.

Thomas and Matsen [20] noted that most patients with recurrent instability could be classified into two groups. The first group of patients was characterized by trauma, unidirectional instability, Bankart lesion, and required surgery (TUBS acronym). The second group was atraumatic, prone to have multidirectional instability and rehabilitation as the first line of treatment. If surgery was needed, a capsular shift would address inferior capsular laxity (AMBRI). This system classifies patients very well at the two opposite ends of the instability spectrum but leaves no room for cases in between or patients that may shift from one condition to the other.

Gerber and Nyffeler [21] proposed a classification system that distinguishes instability from hyperlaxity and recognizes that both can be present independently. Patients are grouped in one of three classes: class A (static instability), class B (dynamic instability), and class C (voluntary dislocations). Patients in class B typically involve the young and active population that we see every day in the clinic where a certain degree of trauma has been involved. This classification assumes that hyperlaxity is not the same as instability, but treatment may vary if combined with instability. A conscious physical exam is crucial to understand if the patient has unidirectional or multidirectional instability with or without hyperlaxity.

The Stanmore classification [22] is a triangular model with three polar types. It was developed to include all varieties of instability in a single framework. Type I is traumatic and structural (resembling TUBS), type II is atraumatic structural (resembling AMBRI), and type III is muscle patterning non-structural instability. The highlight of this classification is that it bears in mind that glenohumeral instability can shift, the patient can be positioned between poles, there is a gradation between traumatic and atraumatic instability, and it incorporates muscle patterning as a distinguished problem.

A physical exam is mandatory no matter what classification system is used. Most importantly, we must determine if there is laxity or instability and the direction of instability. Laxity is a normal finding of glenohumeral translation in asymptomatic individuals, whereas instability is a pathologic excess of translation resulting in symptoms [24]. The most common tests used to determine glenohumeral laxity are the sulcus sign [25], load and shift test [26], anterior and posterior drawer test [27], and the hyperabduction test [28]. They all test for glenohumeral translation in different directions. These tests must be performed bilaterally in all patients. Instability tests are provocative maneuvers. The goal is to reproduce the patient’s instability symptoms and therefore interpret the underlying pathology. The apprehension test [27], and relocation test [29] are described for anterior instability. For posterior instability, the Jerk test may be performed as well as the Kim test for posteroinferior labral lesions [30].

## Principle of management of anterior shoulder instability

After a traumatic anterior shoulder dislocation, the recurrence rate was reported at 14.3–33% in a professional athlete [31]. Several risk factors have been defined for recurrent instability, such as young age, male, athletic activity, and glenoid deficiency [9]. The risk of recurrent instability is significantly higher following non-surgical treatment for these high-risk patients [32–34]. For this reason, the choice of treatment for first episode dislocation has been shifted to primary shoulder stabilization for elite athletes and those with high risks [34].

**Figure 1:**
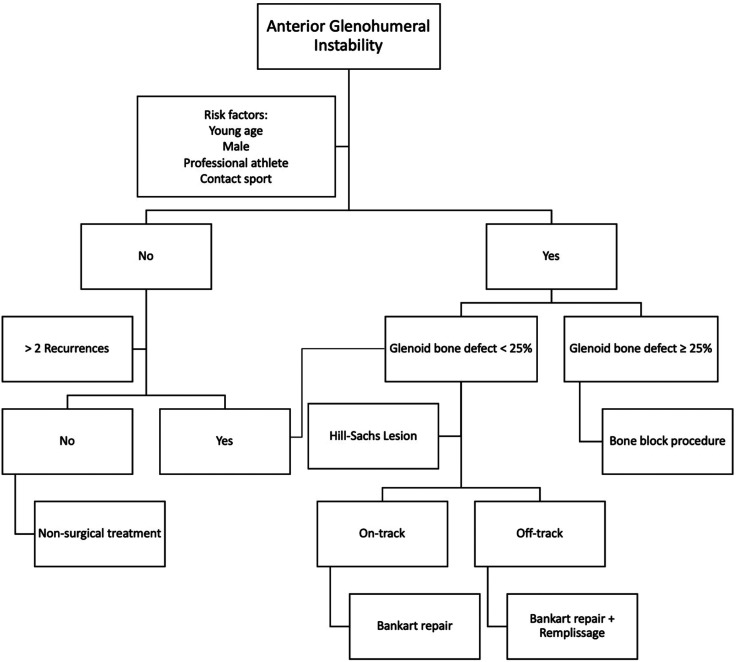
Algorithm for the management of patients with anterior glenohumeral instability.

The decision of surgical treatment should involve the timing of the surgery apart from the careful patient selection. Moreover, detailed information regarding glenoid or humeral bone loss should be sought. Accordingly, a few methods were proposed to quantify the glenoid bone defect, i.e., the face view of reconstructed glenoid CT scan and arthroscopic measurement [35]. The current treatment algorithm is as below (Figure 1).

In the case when risk factors are presented, surgical management is always recommended. The best strategy for surgical management will depend on the presence of a critical glenoid bone defect. Patients with glenoid bone defect < 25% will not require additional procedure when Hill-Sachs lesion does not present.

Currently, there are two options to deal with the first dislocation based on the patient’s risk factors for recurrence instability. Some surgeons recommend early stabilization, but others propose careful rehabilitation followed by immobilization and only proceed with surgery in the case of recurrence. Special attention was given to in-season athletes who sustain a dislocation often offered non-surgical treatment as surgical stabilization does not allow immediate return to play [36].

When performing stabilization surgery, detailed assessment of existing soft tissue injury to the labrum, capsule, glenohumeral ligament, and rotator cuff are mandatory as their presence influences the surgical outcome. Arthroscopic shoulder stabilization is considered the preferred method by most surgeons because of the privilege of allowing detailed inspection on various intraarticular pathologies directly associated with recurrence [37]. For successful arthroscopic Bankart repair [38], it is essential to maintain an optimal glenoid visualization to allow detailed assessment and repair of the capsulo-labral tissue. This could be managed by having an accurate portal establishment and appropriate lateral traction of the proximal arm. A meticulous capsulo-labral release medially from the glenoid neck is crucial to accommodate optimum tissue mobilization and tension. The glenoid bone bed should be prepared with burr/rasp to enhance optimum bone to soft tissue healing. It is recommended to have at least three suture-anchors [9] positioned as low as at the position of 5:30 without excessive medialization to achieve an appropriate labral height [39, 40]. Most importantly, proper tension of the inferior glenohumeral ligament should be restored, which can be achieved by penetrating the suture passer to the more caudal portion of the ligament. Rotator interval closure is still a controversial topic as it is not universally performed for external rotation loss.

The failure rate of a Bankart repair ranges between 5% and 15% at the 6–14 years follow-up with modern suture anchor technique [41]. When dealing with failed arthroscopic Bankart, new significant trauma, surgical errors such as suture anchor malposition, insufficient suture anchor number, failure to re-tension inferior glenohumeral ligament, and failure to address associated pathologies should be investigated. In addition, the surgeon should consider whether the patient had enough rehabilitation time following the index surgery.

Despite the technical evolution of arthroscopic Bankart repair, the open Bankart repair, an anatomic capsulolabral reconstruction described almost 80 years ago, plays an important role, especially for contact athletes. The recurrence rate of arthroscopic Bankart repair was reported up to 26.6% [42] and higher [43] compared to open Bankart repair in contact athletes. The time to recurrence was also observed longer in the open Bankart group compared to the arthroscopic group. As open Bankart surgery can (1) restore capsulolabral complex, (2) retensioning of pathologic capsule by plication, and (3) manage rotator interval lesion, it can resist high stress in collision sports athletes and heavy manual labor [44]. Thus, this can be considered a valuable option between arthroscopic Bankart repair and bone block procedure [11].

## Surgical management of glenoid defects

1. Latarjet procedure

Latarjet-Patte’s open technique, described in 1958 [45], is now widely used to treat significant bone losses in glenohumeral anteroinferior instability. From 1990 to 2000, because of the development of Bankart arthroscopic stabilization, it was not a frequent indication, but it was resumed when the failures related to non-consideration of glenoid and humeral bone losses of the arthroscopic technique were described [46].

The triple effect by which the Latarjet technique works are [47]: the “bone block effect” restoring glenoid bone loss; the “sling effect”, in which the conjoined tendon limits anterior translation in a position of abduction and external rotation; and the “ligament effect” by using the coracoacromial ligament stump to reattach the medial capsule.

The current specific indications for using both the open and arthroscopic Latarjet technique are:

1. Glenoid bone loss from 15% or greater than 25% (subcritical and critical respectively) [48].
2. Patients classified in groups III and IV of Di Giacomo’s algorithm [5].
3. Patients with an Instability Severity Index Score (ISIS) or Glenoid Track. Instability Management Score (GTIMS) greater than 4 points [49].

We can summarize the Latarjet open technique in seven steps: 1. Beach chair position, with a back cushion, 2. Classic deltopectoral approach, 3. Osteotomy of the coracoid, with a minimum of 2 cm in length and subsequent preparation, 4. Horizontal split of the subscapular and vertical capsulotomy, 5. Preparation: creaking the lower anteroinferior surface of the glenoid, 6. Fixing the coracoid with two cortical screws, 7. Closure of the capsule and coracohumeral ligament.

Variants of the technique have been described, such as the Congruent Arc Latarjet [50], in which the coracoid is rotated 90°, placing its inferior surface parallel to the surface of the glenoid. Bhatia and Kandhari [51], a cadaveric and tomographic study, showed that the classic Latarjet corrects glenoid defects from 10 to 25%, having the possibility of increasing the indication with the congruent arch technique up to 40% of glenoid bone loss, therefore it could be considered in major glenoid defects.

The most frequent risk factors for poor results of the Latarjet procedure were clearly identified by Di Giacomo [10]:

1. Atraumatic mechanism of dislocation, which multiplies the possibility of failure of the technique by five.
2. Bilateral dislocation, which multiplies the chance of recurrence by four.
3. Female patients that multiply the chance of failure by three. This is probably related to anatomical factors, such as a smaller glenoid size.

Lafosse and Boyle [52] described the arthroscopic Latarjet procedure, with results like the open technique, with a reported recurrence rate between 2–4.9% and a 98% satisfaction index, although greater residual apprehension is described with the arthroscopic technique, with less reabsorption of the graft. Leuzinger [53] highlighted the long learning curve of the arthroscopic technique, requiring a surgeon to have performed between 20 and 25 procedures to minimize the risk of complications. The graft must be at least 25 mm long by 14 mm wide to perform the technique correctly. A previous fracture or malformation of the coracoid is considered a contraindication.

1. Iliac crest bone graft

The use of a tricortical iliac crest bone graft for managing anterior glenoid bone loss was described over 100 years ago, and this procedure is now synonymous with its authors, Eden [54] and Hybinette [55]. Various iterations have been described, but an open procedure through a subscapularis split securing the bone block with two screws and, more recently, an open and arthroscopic procedure, through the rotator interval, securing the bone block with suture-buttons are the current standard.

Previously, the indications for using an Eden-Hybinette procedure were generally considered to be for patients with a failed coracoid transfer, abnormal coracoid morphology, or particularly large glenoid loss [56]. For more “routine” levels of glenoid bone loss, with or without an engaging Hill-Sachs lesion, considered to require a bone block procedure, a coracoid transfer procedure (Latarjet) has generally been the procedure of choice. Proponents of the Latarjet consider that the additional dynamic sling effect of the conjoint tendon and the requirement of not having to use an iliac crest autograft are favorable aspects over an Eden-Hybinette procedure. However, a coracoid transfer does alter the anatomy and carries a higher risk of potential nerve injury.

**Figure 2:**
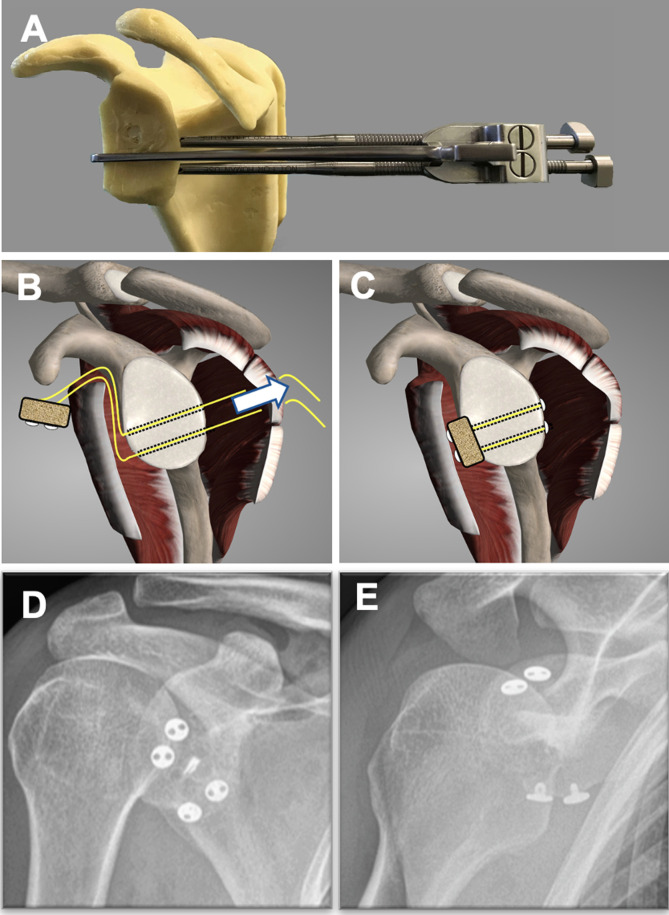
Suture-button Eden-Hybinette.

The recent development of a suture-button fixation technique using a posterior drill guide system that allows for accurate drill tunnel placement and can be used both open and arthroscopically has revived interest in the Eden-Hybinette procedure [57]. The arthroscopic procedure has the advantage over both an open and arthroscopic Latarjet procedure as, due to the flexibility of sutures, it can be undertaken as a purely intra-articular procedure through the rotator interval without compromising subscapularis (Figure 2).

A study in 2018 described the results of 26 patients with recurrent anterior instability with bone loss that underwent an arthroscopic Eden-Hybinette procedure using suture-button fixation [58]. At an average follow-up of 29.6 months (range 24–30) none of the patients had had a re-dislocation with an average Rowe score of 96.4 (SD 6.5) and Walch-Duplay of 93.2 (SD 7.8). The average loss of external rotation, compared to the non-operated side, was 4.40 (SD 8.70), and 92.3% of the grafts had healed on CT scan. Similar techniques using suture-tape cerclage to secure the bone block, avoiding the use of any metalwork, have also been described [59]. Additionally, human allograft and equine xenograft bone blocks have also been used to avoid donor site morbidity [60].

**Figure 3:**
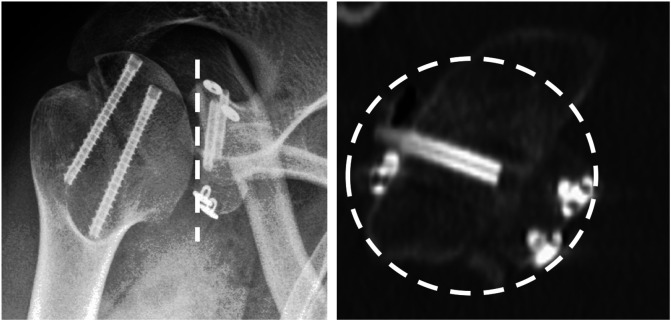
Revision Eden-Hybinette.

In the revision setting, when using the Eden-Hybinette procedure for a failed coracoid transfer, the guided suture-button fixation system has some advantages over the more traditional screw fixation. By undertaking an arthroscopic procedure through the rotator interval only, no dissection of the anterior surface of the subscapularis, where the anatomy will be distorted, or a further split of the subscapularis, which may compromise its function, are required. Additionally, if there is any retained hardware in the glenoid, a “safe trajectory” for the drill-guide and drill holes, which are of a smaller diameter than screws, can be pre-planned to avoid the hardware [61] (Figure 3).

With the evolutions to the Eden-Hybinette procedure that has been described above, there is an increase in its use to treat primary anterior glenoid bone loss. While suture-button fixation can be used for both a Latarjet and an Eden-Hybinette procedure, the ability to undertake the Eden-Hybinette procedure arthroscopically through the rotator interval, without compromising subscapularis or distorting the anatomy, are a significant advantage.

1. Other procedures

Other bone supply alternatives include autologous distal clavicle grafts [62], allographic proximal and distal tibia [63, 64], proximal and distal femur bone grafts [65], according to the surgeon’s choice. They are a good indication of glenoid bone deficits in which the coracoid is insufficient in epileptic patients and as an option to review a failed Latarjet operation [64].

### Distal tibia allograft

This technique has become popular in recent years. The lateral aspect of the distal tibia is used because its curvature is like the curvature of the native glenoid. Provencher et al. [63] reported promising early results in a small case series report in patients with greater than 30% glenoid bone loss. The main advantages of the technique are improved graft availability, the ability to have a cartilaginous interface between the humerus and glenoid graft, and excellent glenoid arc articular conformity [63].

A matched cohort analysis comparing the clinical outcomes between patients undergoing distal tibia allograft procedure with Latarjet technique showed similar outcomes [64].

### Distal clavicle autograft

The anatomy of a distal clavicular graft has several favorable characteristics as a replacement for glenoid bone [62]. The graft provides an articular surface that is congruent with the glenoid, and it has a large surface area for fixation and bony union [62].

Distal clavicle has a broader radius of reconstruction than that of a coracoid graft [66] but, on the other hand, had greater variability and lower density than coracoid grafts [67].

## Surgical management of cephalic defects

Several surgical procedures have been described to treat engaging Hill-Sachs bone defects, including bone graft, retrograde desimpaction, arthroplasty, partial humeral head resurfacing, humeral rotation osteotomy, and remplissage.

Remplissage is a widespread procedure that consists of an arthroscopic posterior capsulodesis and infraspinatus tenodesis to fill the Hill-Sachs lesion in addition to an arthroscopic Bankart repair.

A meta-analysis by Camus et al. [68] suggested that in case of anterior shoulder instability with engaging Hill-Sachs lesion and with up to 20–25% glenoid bone loss, arthroscopic Bankart repair + remplissage reduces recurrent instability by 4-fold comparing with an isolated Bankart repair, with better functional outcomes.

Although the authors of the original description mentioned not having found limitation of postoperative mobility [69], recent studies reported risk of external rotation stiffness in abduction compared with Bankart repair without refill at short-term follow-up [70].

## 3D printing in the treatment of glenohumeral instability

Surgeons are used to working with X-rays, 2D CT scans, and magnetic resonance images to evaluate patients’ anatomy. Spatial 3D rendering was done only in our minds. With emerging 3D renderings, the third dimension could be reproduced and improve the diagnostic of some pathologies and deformities but lack the tactile feeling. 3D printing can add this feeling. This technology is revolutionizing manufacturing processes in industry and medicine.

There are many ways to built anatomical models and surgical guides that may aid surgical execution. Preparing the anatomy for 3D printing can be a demanding task. In some cases, multiple software must be used to prepare and convert files. Some models, due to the particularity of the anatomy, demand special supports making the process a time-consuming task.

**Figure 4:**
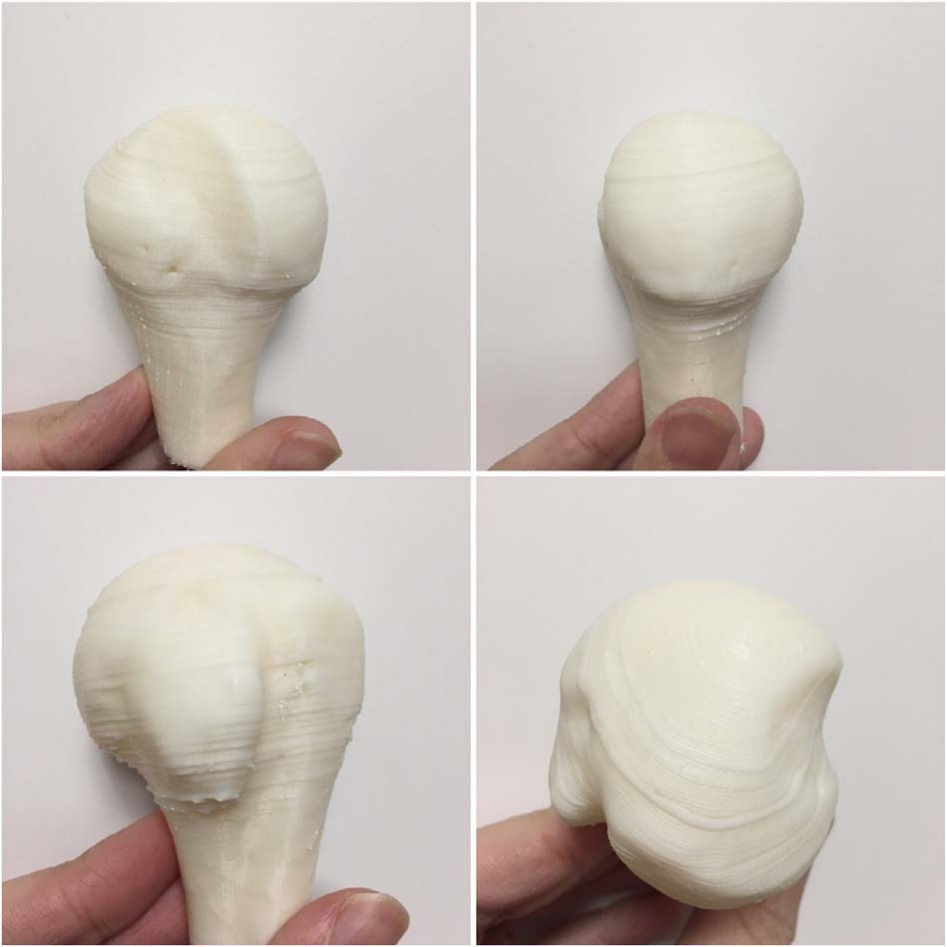
3D Printed model of a humeral head defect.

Three-dimensional technologies can help to evaluate glenoid and humeral defects (Figure 4).

**Figure 5:**
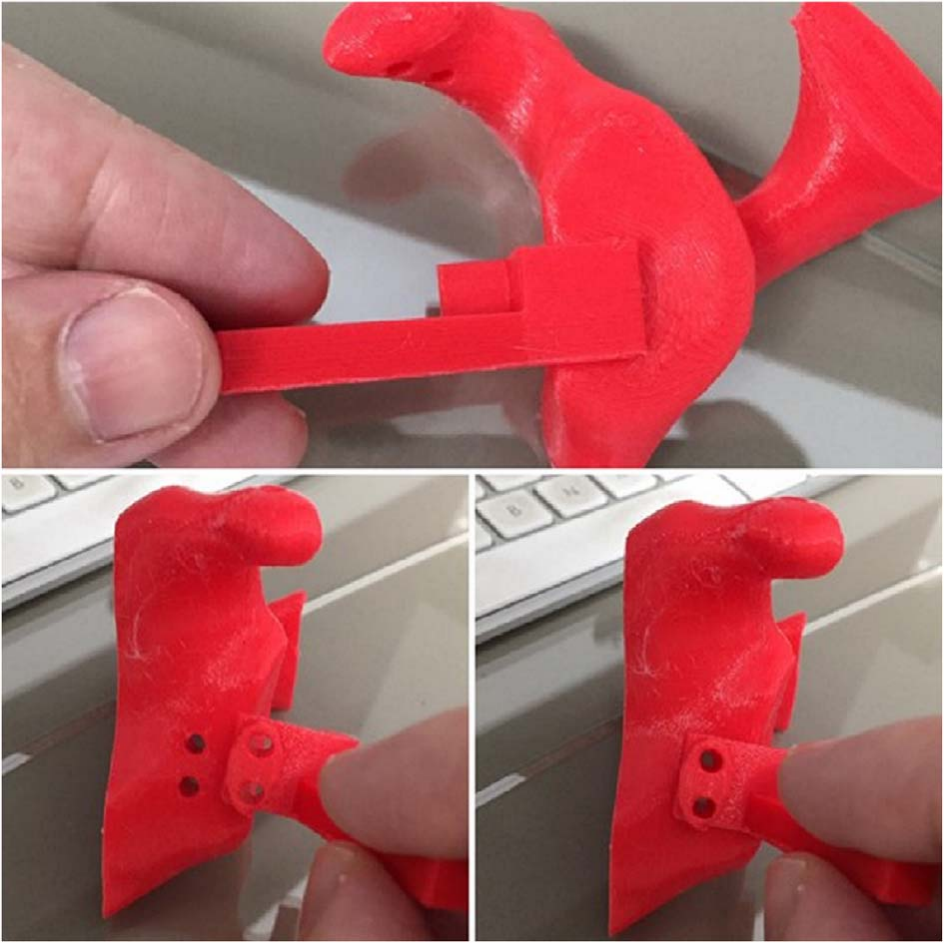
3D Printed model of a glenoid with the location for screws and a 3D printed model of a patient-specific guide to position the screw.

**Figure 6:**
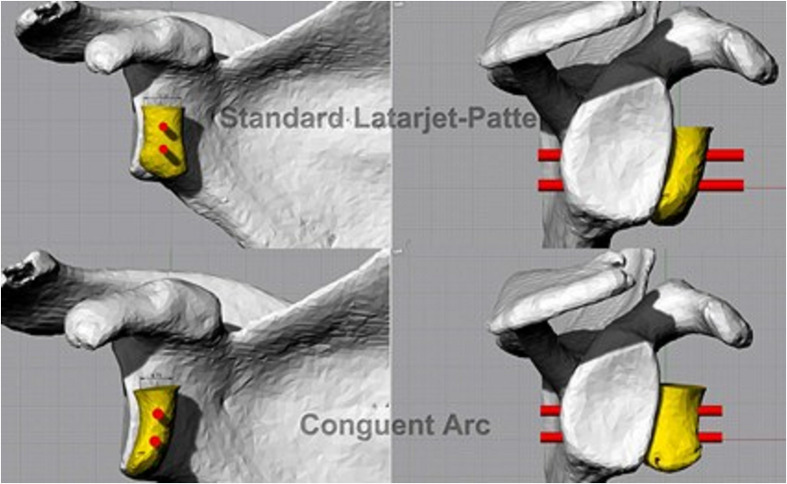
3D digital planning of a Latarjet and Congruent Arc procedure.

Patient-specific guides (Figure 5) are low-cost surgical instruments and can be used in shoulder instability surgery. They allow the surgeon to define the optimal implant location better and accurately execute the surgical plan decreasing errors related to implant malposition (Figure 6).

Three-dimensional printing will undoubtedly become an essential tool to achieve the best results in glenohumeral instability surgery.

## Conflict of interest

The authors declare that there is no conflict of interest.

## Authors contributions

*Daniel Moya*: Coordinated the publication, submitted it, wrote sections: “Surgical management of cephalic defects” and “Other procedures” for surgical management of glenoid defects.

*Nuri Aydin*: Wrote section “Introduction”.

*Nobuyuki Yamamoto*: Wrote section “Biomechanics of shoulder instability”.

*Juan Pablo Simone*: Wrote section “Classification and clinical examination”.

*Paul Patiño Robles*: Wrote Section “Latarjet procedure”.

*Graham Tytherleigh-Strong*: Wrote section “Iliac crest bone graft”.

*Bruno Gobbato*: Wrote section “3D printing in the treatment of glenohumeral Instability”.

*Erica Kholinne*: Wrote section “Principle of management of anterior shoulder Instability”.

*In-Ho Jeon*: Wrote section “Principle of management of anterior shoulder Instability”.

## Funding

This research did not receive any specific funding.

## Ethical approval

Ethical approval was not required.

## Informed consent

This article does not contain any studies involving human subjects.
